# Pneumatosis intestinalis leading to perioperative hypovolemic shock: Case report

**DOI:** 10.1186/1749-7922-6-15

**Published:** 2011-05-08

**Authors:** Yukako Takami, Toshimori Koh, Minoru Nishio, Noboru Nakagawa

**Affiliations:** 1 Department of Surgery, Social Insurance Kobe Central Hospital, 2-1-1, Sohyama-cho, Kita-ku, Kobe City 651-1145, Japan

**Keywords:** pneumatosis intestinalis, surgical management, death, hemorrhage, autopsy

## Abstract

Pneumatosis intestinalis (PI) is an uncommon disorder defined as multiple foci of gas within the intestinal wall. Despite recognition of an increasing number of cases of PI, the optimal management strategy, whether through surgical or other means, remains controversial. The present report describes the case of a patient with PI who underwent exploratory laparotomy without specific findings and who ultimately died due to extensive intestinal hemorrhage that was possibly triggered by surgery.

## Background

Pneumatosis intestinalis (PI) is an uncommon and poorly understood disorder defined by the presence of multiple gas foci within the intestinal wall. In adult patients, PI may be associated with a good prognosis in response to conservative management, but severe cases require surgical management and sometimes result in death. The surgical indications and surgical risks associated with PI have not been definitively established, despite an increasing number of cases. The present report describes the case of a patient with PI who underwent exploratory laparotomy without specific findings and who ultimately developed fulminant intramural intestinal hemorrhage that was possibly triggered by surgery.

## Case presentation

### Case report

**Figure 1:**
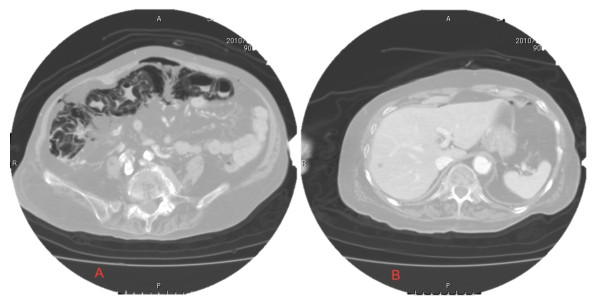
**CT**. Abdominal CT reveals diffuse intramural gas from the ascending colon to the transverse colon and a large amount of free air in the abdominal cavity without portal venous air or extraluminal fluid collections. This study shows diffuse pneumoperitoneum, which led us to suspect the presence of gastrointestinal perforation. Portal venous gas, which frequently follows severe pneumatosis intestinalis, is also absent.

An 81-year-old female nursing home resident presented to our Emergency Department with hematochezia. Past medical history included appendectomy, atrial fibrillation treated with cibenzoline, an 11-year history of rheumatoid arthritis treated with prednisone at 5 mg/day, prior cerebral infarction with ongoing treatment with cilostazol at 200 mg/day, and a percutaneous endoscopic gastrostomy (PEG) established 1 year previously. On arrival, the patient did not show severe status on physical examination and vital signs were within normal limits, including a blood pressure of 130/80 mmHg. Abdominal examination only revealed abdominal distention and mild tenderness in the right upper quadrant, without guarding or rebound tenderness. Bloody stools were observed in her diaper. Noteworthy findings from laboratory evaluation comprised only an elevated white blood cell count (WBC) of 10.6 ×10^3^/μL and mildly elevated C-reactive protein of 1.6 mg/dL. No anemia was apparent, hematocrit was 41.9% and hemoglobin level was 13.5 g/dL. However, computed tomography (CT) revealed diffuse intramural gas from the ascending colon to the transverse colon and a large amount of free air in the abdominal cavity without portal venous air, extraluminal fluid collections or any specific signs indicating ileus or mesenteric artery occlusion (Figure 1). Upper gastrointestinal (GI) endoscopy showed no evidence of perforation in the upper GI tract. Arterial blood gas analysis showed: pH, 7.38; bicarbonate, 24.3 mmol/L; and WBC increased to 11.8 ×10^3^/μL.

**Figure 2:**
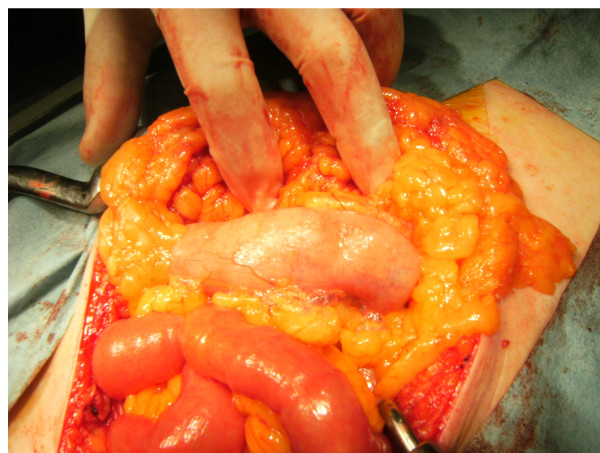
**Intraoperative findings**. Intraoperatively, macroscopic examination of the abdominal cavity shows multiple subserosal bubbles with a diameter of 1-2 mm, mainly around the transverse colon. The appearance of these cystic bubbles is compatible with the characteristics of pneumatosis intestinalis.

Persistence of abdominal symptoms, absence of upper GI perforation, and results from CT strongly suggested lower intestinal perforation and consequent intestinal necrosis. We therefore decided to perform emergent laparotomy. At the beginning of the operation, vital signs remained stable. Observation of the abdominal cavity found no hydroperitoneum, but intraabdominal tissues appeared friable and hemorrhagic. Although the intestine was explored very carefully from the ligament of Treitz to the pouch of Douglas, no indications of gross perforation, ischemia, or tumor were identified. However, multiple subserosal bubbles (diameter, 1-2 mm) were observed, mainly around the transverse colon (Figure 2). During these procedures, the spleen was slightly injured. Although the injury itself was only slight and easy to repair immediately using pressure with oxidized cellulose (Surgicel), bleeding appeared to continue and total blood loss was estimated at 730 mL. Blood pressure decreased to 65/43 mmHg. Hemoglobin and hematocrit decreased markedly to 4.8 g/dL and 15.3%, respectively. Without any gross detection of intestinal perforation, exploratory laparotomy was completed with placement of two Penrose drains within the abdominal cavity, at which point total blood loss was estimated at 1100 mL. Blood pressure was 58/33 mmHg, heart rate was 67 beats/min, and body temperature was 32.9°C. Despite all resuscitation measures including transfusion, the patient died of hypovolemic shock 3 h after closure of the incision. The total amount of blood produced by the drains was 220 mL.

### Autopsy

**Figure 3:**
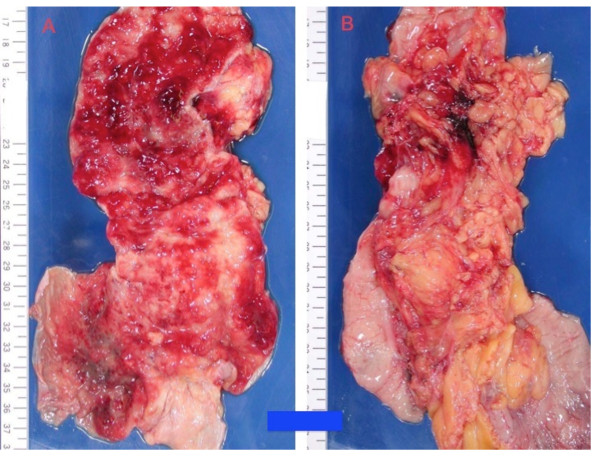
**Macroscopic appearance of the ascending colon**. These pictures show two sides of a specimen of the ascending colon dissected at autopsy (A: mucosal side; B: serous side). The macroscopic appearance of the specimen shows diffuse hemorrhage on both serous and mucosal sides, but a lack of any necrotic feature, consistent with a finding of intraluminal bleeding.

Autopsy was performed at 20 h 25 min after death. A total of 150 mL of hemorrhagic ascites was observed within the abdomen. Diffuse bleeding was apparent around the left diaphragm, and multiple nodular hemorrhages were detected on the greater omentum. The spleen weighed 50 g, with no specific gross abnormalities other than a small amount of bleeding, and the liver weighed 820 g. The PEG tube was without abnormality. No specific findings were noted from the duodenum to the terminal ileum. Multiple emphysematous foci were detected on the serosa and mucosa from the terminal ileum to the descending colon (Figure 3), and a 3-cm hematoma was present on the serosa of the ascending colon. Blood was grossly detected intratubally from the terminal ileum to the descending colon. Diffuse hemorrhagic changes were present horizontally on the mucosal side and to a lesser degree on the serous side, consistent with a finding of intraluminal bleeding. Numerous cystic bubbles, each 1-2 mm in diameter, were present within several layers in vertical specimens of the mucosal layer. No signs of obvious necrotic change or coagulant necrosis were seen within the intestine. On the basis of the autopsy findings, cause of death was determined as hypovolemic shock due to intraluminal hemorrhage from the terminal ileum to the descending colon, with fulminant onset in the perioperative period.

## Discussion

PI is an uncommon condition characterized by the presence of multiple cystic or linear gas deposits within the intestinal wall. In adult patients, PI is frequently asymptomatic and detected only incidentally. DuVernoi first described the condition in 1783. Despite increasing recognition of PI with more prevalent use of CT and colonoscopy, the pathogenesis remains poorly understood, even though the majority of the literature on PI has placed an emphasis on explaining its etiology.

PI is frequently asymptomatic in adults and does not require specific therapy unless abdominal pain, emesis, fever, diarrhea or hematochezia is present. Pneumoperitoneum and pneumoretroperitoneum can be present, but are generally considered as complications rather than causes of PI [1]. Peritonitis may occur, but is uncommon, and perforation is typically absent when only mild clinical symptoms are present [1]. Most reported cases of adult PI detail a benign course in response to conservative management with hyperbaric oxygenation or metronidazole. Death may occur in rare cases, typically associated with severe comorbid conditions (e.g., cancer, immunosuppressed status due to chemotherapy, diabetes mellitus, or portal venous air embolism) [2-5], or acute abdomen followed by bowel ischemia, bowel obstruction, and portal venous gas (PVG) [6]. The cause of death described in fatal PI cases ranges from sepsis to concomitant malignancies, as well as air embolus in the portal vein or colon perforation [2,5,7,8]. To the best of our knowledge, no previous reports have described life-threatening hemorrhage simply due to PI in adults in either the perioperative or non-perioperative period.

Surgical management of PI, usually consisting of urgent laparotomy in patients with acute abdomen, remains controversial. While surgery is probably necessary in severe cases, routine utilization of surgical management may be associated with poor prognosis. This determination is complicated by the fact that most studies of PI have described etiology or radiographic findings, but few have addressed clinical management, particularly from a surgical perspective [9-11]. Knechtle evaluated 27 patients with PI and reported the highest mortality rate among PI patients with bowel ischemia who underwent surgery, demonstrating associations of low pH (<7.3), low serum bicarbonate (<20 mmol/L) and elevated serum lactic acid (LA) (>2 mmol/L) with ischemic bowel and mortality [9]. Hawn et al. assessed 86 patients showing PI on CT and reported a mortality rate of 73% among patients with complicated ischemic bowel and 83% in patients with hepatic failure [10]. These investigators identified elevated serum LA (>2.0 mmol/L) as a predictor of mortality and recommended urgent surgical intervention in patients with elevated LA [9,10]. Further, they reported that cystic emphysema was associated with favorable prognosis, while linear emphysema was associated with poor prognosis [9,10], and described CT findings of focal thickening, dilated or fluid-filled bowel segments, portal or mesenteric venous gas, and thrombi in the superior mesenteric artery [10]. Greenstein suggested that indications for surgical management include WBC >12×10^3^/μL, and/or emesis, particularly in patients >60 years old [11]. He also reported that patients with sepsis at the time of PI diagnosis were at high risk for mortality regardless of whether surgical management was performed [11]. Interestingly, although the combination of PI and PVG has previously been reported to show a high mortality rate, surgically treated PI patients with PVG showed a slightly decreased risk of death in that report [11].

In the present case, intestinal perforation was suspected due to the presence of pneumoperitoneum on CT. Laparotomy revealed gross PI without any macroscopic intestinal perforation. In retrospect, the present case satisfied the surgical indications detailed in previous studies, although LA was not assessed in our patient. Of note, metabolic acidosis was not present preoperatively, explaining the absence of bowel ischemia and consequent sepsis.

In terms of the relationship between PI and hemorrhage, some reports have described adult PI presenting with hematochezia. However, most of those reports described a benign course, and the present case appears to represent the first report of an adult patient with PI who developed intraluminal hemorrhage resulting in hypovolemic shock and death in the perioperative period. Discussion of the factors that may have contributed to bleeding is important in this case. For example, cilostazol can increase the risk of bleeding, and prednisone can cause disruption of gastrointestinal tissues, both of which may have increased the risk of GI compromise. However, weights of the spleen and liver were within normal limits, and hemorrhage was localized to the described areas of the colon, suggesting that bleeding was not due to splenic and/or endoceliac bleeding caused by splenic injury or other complications during the laparotomy. To discuss the origin of the hemorrhage in greater detail, body weight of the patient was approximately 40 kg, preoperative hematocrit was 41.9% and hematocrit after the rapid hemorrhage was 15.3%. According to the Gross formula, acute blood loss = blood volume × [Hct(i) - Hct(f)]/Hct(m), where Hct(i), Hct(f) and Hct(m) were the initial, final and mean (of initial and final values) hematocrits, respectively. In the current case, acute blood loss was calculated as approximately 2600 mL. Intraoperatively assessed blood loss from the abdominal cavity was 1100 mL, including the splenic bleeding. Postoperatively, platelets decreased to 80×10^3^/μL from the preoperative level of 182 ×10^3^/μL. The postoperative platelet level may indicate occurrence of disseminated intravascular coagulation (DIC), but because postoperative laboratory data obtained before death only examined complete blood cell count, our ability to evaluate the existence of DIC was limited. Furthermore, the patient presented with hematochezia from admission, at which point she presented with neither abnormal vital signs nor anemia. Spontaneous intestinal bleeding could be assumed to have continued during the whole clinical course from admission until death. Furthermore, given the lack of intraoperative colonoscopy, it is difficult to completely exclude the possibility of rough manipulation of the bowel causing the severe hemorrhage. In addition to the etiology of PI remaining unclear, clear explanation for the intestinal bleeding in the current case is difficult to provide. However, the previously stable blood pressure, hemoglobin and hematocrit all rapidly and substantially decreased only right after the slight injury to the spleen, 2 h after the incision and lysis of adhesions of the whole lower intestine had already been finished without encountering any problems. On the basis of this fact, we concluded that intestinal hemorrhage leading to hypovolemic shock was due to the rupture of pneumatosis accelerated by some molecular factors released following splenic injury, rather than simply the splenic bleeding itself. Although the pathophysiological process underlying PI remains poorly understood, we speculate that some molecular factors released during surgical intervention, particularly after partial injury of the spleen, accelerated rupture of the submucosal emphysema followed by intraluminal hemorrhage.

## Conclusion

This represents a rare case of PI that initially presented in benign fashion before progressing rapidly to a fulminant and fatal course. Had the bleeding lesion been clearly identified, complete resection could have been performed during laparotomy and may have resulted in a different outcome.

PI is frequently asymptomatic in adults and detected incidentally. The true incidence of PI is thus likely much higher than appreciated. The present case serves as an illustrative example of the risk of surgical management in patients with PI. Surgeons should recognize that surgery may induce rupture of intestinal pneumatosis.

## Abbreviations

PI: pneumatosis intestinalis; PEG: percutaneous endoscopic gastrostomy; WBC: white blood cell; CT: computed tomography; GI: gastrointestinal; PVG: portal venous gas; LA: lactic acid; DIC: disseminated intravascular coagulation.

## Competing interests

The authors declare that they have no competing interests.

## Authors' contributions

YT participated in the care of this patient and observed the autopsy, conducted a search of the literature, and authored this manuscript. TK participated in the care of this patient and provided editorial commentary. MN participated in the care of this patient. NN is the chief director of the Department of Surgery and oversaw the editing process.

All authors have read and approved the submitted version of the manuscript.

## Consent

Written informed consent for publication of this case report and all accompanying images was obtained from the patient's next of kin. A copy of the written informed consent is available for review.

## Figures and Tables

**Figure 4:**
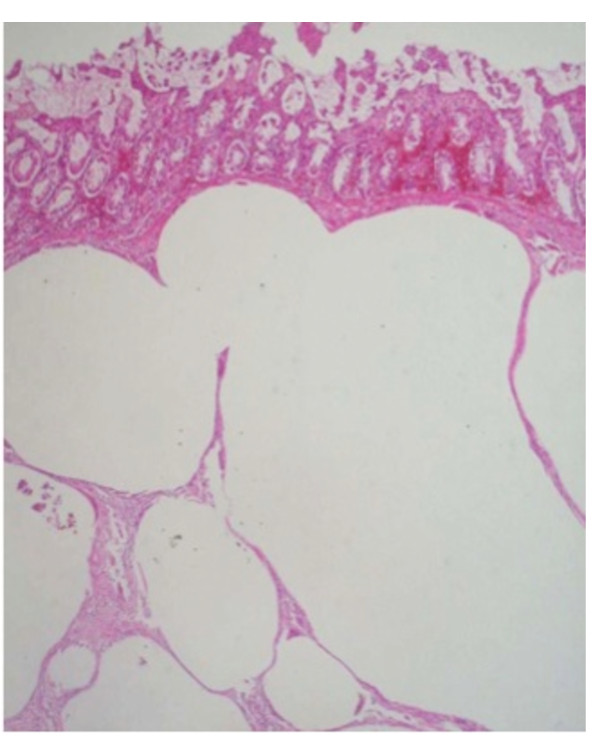
**Microscopic histological appearance of the ascending colon**. Microscopic histological appearance of the specimen of the ascending colon shows multiple foci of pneumatosis, which are compatible with pneumatosis cystoides intestinalis. This study also shows hemorrhage within the mucosa without any necrotic features.
